# *catena*-Poly[[diaquazinc(II)]-μ-*trans*-4,4′-diazenediyldibenzoato-κ^4^ *O*,*O*′:*O*′′,*O*′′′]

**DOI:** 10.1107/S1600536809012604

**Published:** 2009-04-10

**Authors:** Bo Liu, Qiang Xu

**Affiliations:** a National Institute of Advanced Industrial Science and Technology (AIST), Ikeda, Osaka 563-8577, Japan, and Graduate School of Science and Technology, Kobe University, Nada Ku, Kobe, Hyogo 657-8501, Japan

## Abstract

The title compound, [Zn(C_14_H_8_N_2_O_4_)(H_2_O)_2_]_*n*_, consists of zigzag chains of Zn atoms bridged by azobenzene-4,4′-dicarboxyl­ate ligands. The Zn^II^ atom, lying on a twofold rotation axis, is coordinated by four O atoms from the carboxyl­ate groups and two water mol­ecules, giving rise to a considerably distorted octa­hedral coordination envionment. The ligand lies on an inversion center. In the crystal structure, π–π inter­actions between the ligands [inter­planar distance = 3.527 (3) Å] assemble the chains into a sheet-like structure. O—H⋯O hydrogen bonds between the coordinated water mol­ecules and carboxyl­ate O atoms connect the sheets into a three-dimensional network.

## Related literature

For related structures, see: Chen *et al.* (2008 ▶); Bai *et al.* (2008 ▶); Mukherjee *et al.* (2004 ▶); Reineke *et al.* (2000 ▶).
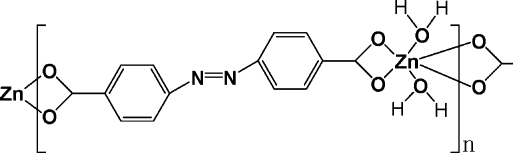


## Experimental

### 

#### Crystal data

[Zn(C_14_H_8_N_2_O_4_)(H_2_O)_2_]
*M* *_r_* = 369.63
Monoclinic, 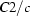

*a* = 22.392 (5) Å
*b* = 4.9308 (10) Å
*c* = 12.185 (2) Å
β = 90.30 (3)°
*V* = 1345.3 (5) Å^3^
*Z* = 4
Mo *K*α radiation
μ = 1.86 mm^−1^
*T* = 293 K
0.14 × 0.09 × 0.08 mm

#### Data collection

Rigaku R-AXIS RAPID diffractometer
Absorption correction: multi-scan (*ABSCOR*; Higashi, 1995 ▶) *T*_min_ = 0.781, *T*_max_ = 0.865
6205 measured reflections
1532 independent reflections
1194 reflections with *I* > 2σ(*I*)
*R*_int_ = 0.043

#### Refinement

*R*[*F* ^2^ > 2σ(*F* ^2^)] = 0.044
*wR*(*F* ^2^) = 0.109
*S* = 1.04
1532 reflections
110 parameters
8 restraints
H atoms treated by a mixture of independent and constrained refinement
Δρ_max_ = 1.37 e Å^−3^
Δρ_min_ = −0.89 e Å^−3^

### 

Data collection: *PROCESS-AUTO* (Rigaku, 1998 ▶); cell refinement: *PROCESS-AUTO*; data reduction: *PROCESS-AUTO*; program(s) used to solve structure: *SHELXS97* (Sheldrick, 2008 ▶); program(s) used to refine structure: *SHELXL97* (Sheldrick, 2008 ▶); molecular graphics: *Mercury* (Macrae *et al.*, 2006 ▶); software used to prepare material for publication: *publCIF* (Westrip, 2009 ▶).

## Supplementary Material

Crystal structure: contains datablocks I, New_Global_Publ_Block. DOI: 10.1107/S1600536809012604/hy2186sup1.cif

Structure factors: contains datablocks I. DOI: 10.1107/S1600536809012604/hy2186Isup2.hkl

Additional supplementary materials:  crystallographic information; 3D view; checkCIF report

## Figures and Tables

**Table 1:** Selected bond lengths (Å)

Zn1—O3	1.985 (3)
Zn1—O2	2.572 (2)
Zn1—O1	1.995 (2)

**Table 2:** Hydrogen-bond geometry (Å, °)

*D*—H⋯*A*	*D*—H	H⋯*A*	*D*⋯*A*	*D*—H⋯*A*
O3—H3*A*⋯O1^i^	0.82	1.92	2.735 (4)	171
O3—H3*B*⋯O2^ii^	0.81 (6)	1.91 (6)	2.712 (4)	173 (6)

Symmetry codes: (i) 
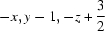
; (ii) 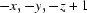.

**Fig. 1.**
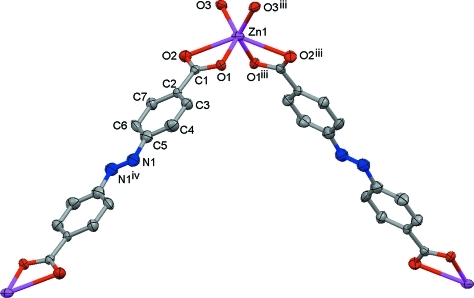
Part of the chain in the title compound. Displacement ellipsoids are drawn at the 50% probability level. H atoms have been omitted for clarity. [Symmetry codes: (iii) -x, y, 3/2 - z; (iv) 1/2 - x, 5/2 - y, 1 - z.]

**Fig. 2.**
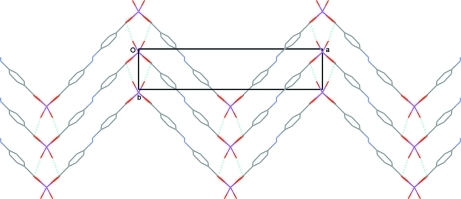
The crystal packing diagram showing hydrogen bonds (dashed lines).

## supplementary crystallographic information

### Comment

Metal-organic frameworks, in which metal ions are bridged by organic ligands, have a rapid development in the past decade due to the various topologies and wide applications. Azodibenzoate-based ligands are a kind of typical aromatic carboxylate ligand employed in the generation of coordination networks, including azobenzene-4,4'-dicarboxylate and azobenzene-3,3'-dicarboxylate and so on. A series of coordination polymers have been reported by employing these ligands as linkers (Chen *et al.*, 2008; Bai *et al.*, 2008; Mukherjee *et al.*, 2004; Reineke *et al.*, 2000). The title compound (Fig. 1 and Table 1) is an analogue to the reported compound [Cd(C_14_H_8_N_2_O_4_)(H_2_O)_2_]_n_ (Chen *et al.*, 2008). The space group of the reported Cd complex is *P*2_1_/*m*, while that of the title compound is determined to be *C*2/*c*. The seperation between the bridged Zn atoms is 17.211 (1) Å and the angle for the three neighboring Zn atoms is 95.84 (8)°. In comparison, the O_aq_···O_carboxylate_ distances for hydrogen bonds are 2.712 (4) and 2.735 (4) Å in the title compound (Table 2), while they are greater than 3.19 Å in the Cd complex. Face-to-face π–π interaction between the ligands exists in the title compound [interplane distance = 3.527 (3) Å], which assembles the Zn–ligand chains into a sheet. O—H···O hydrogen bonds between the coordinated water molecules and carboxylate O atoms connect the sheets into a three-dimensional network (Fig. 2).

### Experimental

The single crystals of the title compound were obtained by solvothermal reaction of Zn(NO_3_)_2_.6H_2_O (0.013 g) and azobenzene-4,4'-dicarboxylic acid (0.007 g) in a mixed solvent of DMSO and H_2_O (20 ml, volume ratio 1:1) at 393 K for 72 h.

### Refinement

Aromatic H atoms were positioned geometrically and refined as riding, with C—H = 0.93 Å and with *U*_iso_(H) = 1.2*U*_eq_(C). H atoms of water molecule were located on a difference Fourier map. One (H3A) of the two water H atoms was fixed with O—H = 0.82 Å and *U*_iso_(H) = 1.5*U*_eq_(O). The other H atom (H3B) was refined isotropically, with a distance restraint O—H = 0.82 (1) Å. In the final difference map, the highest peak and deepest hole were found to be 0.81 and 0.60 Å from N1 atom, respectively.

### Crystal data

**Table:**

[Zn(C_14_H_8_N_2_O_4_)(H_2_O)_2_]	*F*(000) = 752
*M**_r_* = 369.63	*D*_x_ = 1.825 Mg m^−^^3^
Monoclinic, *C*2/*c*	Mo *K*α radiation, λ = 0.71073 Å
Hall symbol: -C 2yc	Cell parameters from 1194 reflections
*a* = 22.392 (5) Å	θ = 3.3–27.5°
*b* = 4.9308 (10) Å	µ = 1.86 mm^−^^1^
*c* = 12.185 (2) Å	*T* = 293 K
β = 90.30 (3)°	Needle, orange
*V* = 1345.3 (5) Å^3^	0.14 × 0.09 × 0.08 mm
*Z* = 4	

### Data collection

**Table:**

Rigaku R-AXIS RAPID diffractometer	1532 independent reflections
Radiation source: rotating anode	1194 reflections with *I* > 2σ(*I*)
graphite	*R*_int_ = 0.043
ω scans	θ_max_ = 27.5°, θ_min_ = 3.3°
Absorption correction: multi-scan (*ABSCOR*; Higashi, 1995)	*h* = −28→28
*T*_min_ = 0.781, *T*_max_ = 0.865	*k* = −6→6
6205 measured reflections	*l* = −14→15

### Refinement

**Table:**

Refinement on *F*^2^	Primary atom site location: structure-invariant direct methods
Least-squares matrix: full	Secondary atom site location: difference Fourier map
*R*[*F*^2^ > 2σ(*F*^2^)] = 0.044	Hydrogen site location: inferred from neighbouring sites
*wR*(*F*^2^) = 0.109	H atoms treated by a mixture of independent and constrained refinement
*S* = 1.04	*w* = 1/[σ^2^(*F*_o_^2^) + (0.0399*P*)^2^ + 7.8756*P*] where *P* = (*F*_o_^2^ + 2*F*_c_^2^)/3
1532 reflections	(Δ/σ)_max_ < 0.001
110 parameters	Δρ_max_ = 1.37 e Å^−^^3^
8 restraints	Δρ_min_ = −0.89 e Å^−^^3^

### Fractional atomic coordinates and isotropic or equivalent isotropic displacement parameters (Å^2^)

**Table:**

	*x*	*y*	*z*	*U*_iso_*/*U*_eq_	
Zn1	0.0000	0.08046 (11)	0.7500	0.0287 (2)	
O1	0.06809 (11)	0.3407 (5)	0.7428 (2)	0.0270 (6)	
O2	0.04382 (12)	0.2669 (5)	0.5705 (2)	0.0343 (6)	
C1	0.07463 (15)	0.3874 (7)	0.6402 (3)	0.0233 (7)	
O3	−0.03292 (14)	−0.2000 (5)	0.6496 (2)	0.0364 (7)	
H3A	−0.0458	−0.3274	0.6857	0.055*	
C2	0.12028 (14)	0.5963 (7)	0.6090 (3)	0.0229 (7)	
C6	0.16404 (19)	0.9039 (8)	0.4779 (4)	0.0401 (10)	
H6	0.1651	0.9768	0.4075	0.048*	
C5	0.20375 (18)	0.9943 (8)	0.5579 (4)	0.0399 (7)	
C7	0.12236 (18)	0.7023 (8)	0.5038 (3)	0.0332 (8)	
H7	0.0960	0.6394	0.4503	0.040*	
C3	0.16064 (17)	0.6897 (8)	0.6873 (3)	0.0323 (8)	
H3	0.1599	0.6179	0.7578	0.039*	
C4	0.20196 (18)	0.8879 (8)	0.6617 (4)	0.0399 (10)	
H4	0.2286	0.9492	0.7150	0.048*	
H3B	−0.037 (2)	−0.207 (12)	0.584 (5)	0.068 (18)*	
N1	0.25044 (16)	1.2046 (7)	0.5460 (3)	0.0415 (7)	

### Atomic displacement parameters (Å^2^)

**Table:**

	*U*^11^	*U*^22^	*U*^33^	*U*^12^	*U*^13^	*U*^23^
Zn1	0.0257 (3)	0.0176 (3)	0.0429 (4)	0.000	0.0010 (2)	0.000
O1	0.0319 (14)	0.0247 (12)	0.0244 (13)	−0.0017 (10)	0.0061 (10)	0.0034 (10)
O2	0.0348 (15)	0.0331 (14)	0.0351 (15)	−0.0108 (12)	0.0020 (12)	−0.0059 (12)
C1	0.0230 (16)	0.0196 (16)	0.0274 (18)	0.0033 (13)	0.0045 (13)	−0.0015 (14)
O3	0.059 (2)	0.0231 (13)	0.0273 (16)	−0.0099 (13)	0.0025 (14)	0.0009 (11)
C2	0.0223 (16)	0.0206 (15)	0.0258 (17)	−0.0011 (13)	0.0080 (13)	−0.0001 (14)
C6	0.045 (2)	0.035 (2)	0.041 (2)	0.0047 (19)	0.0167 (19)	0.0171 (19)
C5	0.0342 (15)	0.0342 (15)	0.0513 (18)	0.0033 (13)	0.0110 (14)	0.0006 (14)
C7	0.038 (2)	0.034 (2)	0.028 (2)	−0.0023 (17)	0.0022 (16)	0.0028 (16)
C3	0.0288 (19)	0.0323 (19)	0.036 (2)	−0.0057 (15)	0.0039 (16)	−0.0006 (17)
C4	0.0274 (19)	0.037 (2)	0.056 (3)	−0.0095 (17)	0.0043 (18)	−0.0082 (19)
N1	0.0368 (15)	0.0375 (15)	0.0505 (17)	0.0050 (13)	0.0094 (14)	0.0012 (13)

### Geometric parameters (Å, °)

**Table:**

Zn1—O3	1.985 (3)	C2—C3	1.389 (5)
Zn1—O3^i^	1.985 (3)	C6—C5	1.389 (6)
Zn1—O2	2.572 (2)	C6—C7	1.401 (5)
Zn1—O1	1.995 (2)	C6—H6	0.9300
Zn1—O1^i^	1.995 (2)	C5—C4	1.371 (6)
O1—C1	1.280 (4)	C5—N1	1.480 (5)
O2—C1	1.243 (4)	C7—H7	0.9300
C1—C2	1.501 (5)	C3—C4	1.383 (5)
O3—H3A	0.8200	C3—H3	0.9300
O3—H3B	0.81 (6)	C4—H4	0.9300
C2—C7	1.385 (5)	N1—N1^ii^	1.207 (7)
			
O3—Zn1—O3^i^	91.71 (17)	C5—C6—C7	119.6 (4)
O3—Zn1—O1	134.60 (11)	C5—C6—H6	120.2
O3^i^—Zn1—O1	101.17 (12)	C7—C6—H6	120.2
O3—Zn1—O1^i^	101.17 (12)	C4—C5—C6	120.2 (4)
O3^i^—Zn1—O1^i^	134.60 (11)	C4—C5—N1	112.5 (4)
O1—Zn1—O1^i^	99.95 (15)	C6—C5—N1	127.3 (4)
C1—O1—Zn1	104.5 (2)	C2—C7—C6	120.2 (4)
C1—O2—Zn1	78.6 (2)	C2—C7—H7	119.9
O2—C1—O1	121.0 (3)	C6—C7—H7	119.9
O2—C1—C2	122.1 (3)	C4—C3—C2	120.9 (4)
O1—C1—C2	116.9 (3)	C4—C3—H3	119.6
Zn1—O3—H3A	109.5	C2—C3—H3	119.6
Zn1—O3—H3B	133 (4)	C5—C4—C3	120.1 (4)
H3A—O3—H3B	117.4	C5—C4—H4	120.0
C7—C2—C3	119.0 (3)	C3—C4—H4	120.0
C7—C2—C1	121.3 (3)	N1^ii^—N1—C5	110.0 (5)
C3—C2—C1	119.7 (3)		
			
O3—Zn1—O1—C1	28.2 (3)	C3—C2—C7—C6	−1.1 (6)
O3^i^—Zn1—O1—C1	131.9 (2)	C1—C2—C7—C6	177.8 (3)
O1^i^—Zn1—O1—C1	−88.5 (2)	C5—C6—C7—C2	0.8 (6)
Zn1—O1—C1—O2	−3.7 (4)	C7—C2—C3—C4	0.8 (6)
Zn1—O1—C1—C2	175.3 (2)	C1—C2—C3—C4	−178.0 (3)
O2—C1—C2—C7	12.6 (5)	C6—C5—C4—C3	0.1 (6)
O1—C1—C2—C7	−166.5 (3)	N1—C5—C4—C3	179.6 (4)
O2—C1—C2—C3	−168.6 (3)	C2—C3—C4—C5	−0.4 (6)
O1—C1—C2—C3	12.3 (5)	C4—C5—N1—N1^ii^	−178.8 (4)
C7—C6—C5—C4	−0.3 (6)	C6—C5—N1—N1^ii^	0.7 (7)
C7—C6—C5—N1	−179.8 (4)		

Symmetry codes: (i) −*x*, *y*, −*z*+3/2; (ii) −*x*+1/2, −*y*+5/2, −*z*+1.

### Hydrogen-bond geometry (Å, °)

**Table:**

*D*—H···*A*	*D*—H	H···*A*	*D*···*A*	*D*—H···*A*
O3—H3A···O1^iii^	0.82	1.92	2.735 (4)	171
O3—H3B···O2^iv^	0.81 (6)	1.91 (6)	2.712 (4)	173 (6)

Symmetry codes: (iii) −*x*, *y*−1, −*z*+3/2; (iv) −*x*, −*y*, −*z*+1.
